# A comparison of theacrine and methylliberine with caffeine as salivary markers for determining gastric emptying

**DOI:** 10.1016/j.ijpx.2025.100442

**Published:** 2025-11-08

**Authors:** Toni Wildgrube, Stefan Senekowitsch, Robin Krüger, Fabian Winter, Michael Grimm, Werner Weitschies, Philipp Schick

**Affiliations:** Department of Biopharmaceutics and Pharmaceutical Technology, Center of Drug Absorption and Transport, University of Greifswald, Germany

**Keywords:** Gastric emptying, Fasted state, In vivo study, Caffeine, Methylliberine, Theacrine, Salivary tracer technique

## Abstract

Gastric emptying is a critical determinant of the pharmacokinetics of orally administered drugs. A key limitation in studies using caffeine as a marker for gastric emptying is the requirement for prior caffeine abstinence, which can complicate study design and participant recruitment. The present study was conducted with the objective of evaluating the potential use of the methylurates methylliberine and theacrine as novel salivary markers for the assessment of gastric emptying of non-caloric liquids, in comparison to the established marker caffeine. A Salivary Tracer Technique (STT) was employed in a crossover study that involved twelve healthy volunteers. The subjects participating in the study were under fasted state conditions according to the guidelines of the Food and Drug Administration (FDA) and the European Medicines Agency (EMA) when they received ice capsules containing either caffeine, methylliberine, theacrine, or a combination of these three substances. Salivary samples were collected at predetermined intervals over 24 h in order to analyse their pharmacokinetic profiles. The results demonstrated a rapid absorption, with maximum salivary concentrations (c_max_) reached within 30 min for all markers. It is noteworthy that methylliberine and theacrine exhibited strong correlations with caffeine in their absorption profiles, with Pearson correlation coefficients of *r* = 0.9973 and *r* = 0.9865, respectively, during the initial 40 min post-administration. Furthermore, the elimination half-lives (t_1/2_) of methylliberine and theacrine were found to differ significantly, with methylliberine exhibiting a rapid elimination profile (t_1/2_ = 1.15 ± 0.12 h) in comparison to theacrine (t_1/2_ = 21.00 ± 7.55 h). These results support methylliberine and theacrine as promising non-invasive markers of gastric emptying, offering viable alternatives to caffeine that may eliminate the need for abstinence and may allow for more efficient multi-tracer pharmacokinetic studies.

## Graphical abstract

**Figure:**
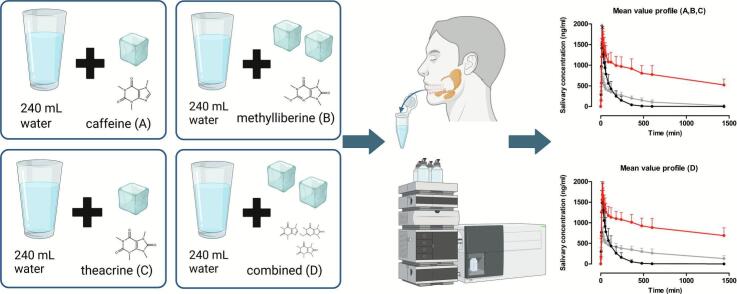

## 1 Introduction

**Fig. 1:**
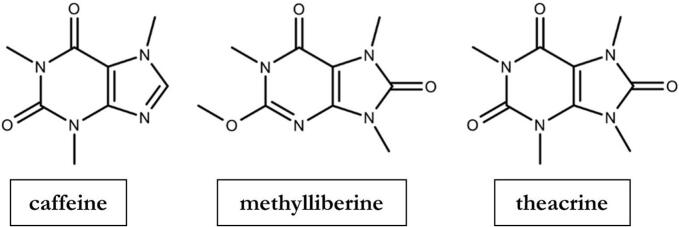
Structural formulas of caffeine, methylliberine and theacrine.

Gastric emptying plays a central role in the pharmacokinetics of orally administered drugs as it regulates their passage from the non-absorbing stomach into the small intestine as the main site of absorption and thus influences their rate of absorption and subsequent systemic availability (Martinez and Amidon, 2002; Kimura and Higaki, 2002; Stillhart et al., 2020; Štefanič et al., 2012). Disruptions or variations in gastric emptying can therefore have a significant impact on the efficacy and safety of oral drug therapy. Consequently, a range of methods have been developed to accurately monitor and assess gastric emptying (Senekowitsch et al., 2022; Hens et al., 2017). Methods for determining gastric emptying range from breath tests to advanced imaging techniques, including scintigraphy and magnetic resonance imaging (Ghoos et al., 1993; Schwizer et al., 2006; Stacher and Bergmann, 1992). While these imaging modalities provide precise and direct assessments, they are resource-intensive and time-consuming, requiring significant financial investment, specialized equipment as well as trained personnel. In contrast, pharmacokinetic markers have emerged as effective alternatives. These markers operate on the principle that gastric emptying is the rate-limiting step in their absorption process, with their detection in blood or saliva serving as an indirect measure to evaluate the rate and kinetics of gastric emptying. Given the invasive nature of blood sampling, saliva has emerged as a simple and non-invasive surrogate matrix for pharmacokinetic assessments, a method known as the Salivary Tracer Technique (STT) (Sager et al., 2018; Grimm et al., 2023a; Eaimtrakarn et al., 2002). However, the applicability of this approach is limited to substances with adequate solubility and permeability, for which a significant correlation between saliva and plasma concentrations can be demonstrated. Building on this principle, compounds such as paracetamol and caffeine have been used as examples of markers in the STT for evaluating gastric emptying of fluids (Grimm et al., 2023a; Zylber-Katz et al., 1984; Glynn and Bastain, 2011; Heading et al., 1973; Newton et al., 1981). Caffeine, in particular, has been extensively employed due to its classification as a food additive by the FDA, which ensures its safe administration in healthy subjects (*EFSA J.*, 2015). As a BCS Class I compound with high solubility and permeability, its absorption is limited primarily by the rate of gastric emptying. Additionally, a well-documented and consistent saliva-to-plasma concentration ratio (s/p ratio) allows caffeine to be measured reliably in saliva (Sager et al., 2018; Newton et al., 1981). However, the ubiquitous presence of caffeine in the diet poses several challenges for its application in the STT. Participants are typically required to abstain from caffeine consumption prior to study enrollment to avoid physiological baseline caffeine levels, which can complicate data interpretation. Alternatively, isotopically labelled substances such as ^13^C_3_-caffeine are employed to distinguish exogenous from study-related caffeine, but these compounds are costly and may limit the feasibility of largescale studies (Grimm et al., 2023b). As shown in Fig. 1, the two methylurates, methylliberine and theacrine, are structurally related to caffeine. However, unlike caffeine, these compounds are found exclusively in selected plants, such as the leaves of certain *Coffea specie*s and *Camellia assamica* var. *kucha*, that are not part of the typical Western diet (Zheng et al., 2002). Nonetheless, the safe oral application of methylliberine and theacrine has been demonstrated in several studies (VanDusseldorp et al., 2020; Cintineo et al., 2022; Wang et al., 2020). Given their structural similarity to caffeine, these substances warrant evaluation as potentially effective alternatives in the STT. Due to this similarity, it is also assumed that methylliberine and theacrine are secreted into saliva in a comparable manner. A rapid initial increase in their salivary concentrations would, as with caffeine, indicate rapid gastric emptying and absorption. When using the salivary tracer technique, contamination of saliva with the marker substance itself must be strictly avoided in order to ensure valid concentration measurements reflecting systemic absorption. To address this issue, Sager et al. introduced a method of encapsulating marker substances in specially prepared ice capsules, which melt rapidly in the stomach and thereby minimize oral contamination (Sager et al., 2018). Notably, the incorporation of methylliberine and theacrine may obviate the necessity for participants to abstain from caffeine prior to study enrollment, thereby facilitating study protocols and enhancing participant compliance. Furthermore, the identification of additional pharmacokinetic markers would present significant advantages, such as the potential to label a pharmaceutical formulation with multiple markers, which would enable a broader application of this technique.

The objective of our study was to quantify methylliberine and theacrine in human saliva and to compare their absorption profiles with that of caffeine following oral administration in the fasted state. To this end, we employed the aforementioned technique described by Sager et al., to prevent oral contamination (Sager et al., 2018). The administration of caffeine, methylliberine, and theacrine was conducted both, individually and combined, to gain a deeper understanding of the pharmacokinetics of each substance. It was anticipated that the resulting salivary profiles would provide valuable insights into the suitability of methylliberine and theacrine for determining gastric emptying of non-caloric liquids in the fasted state.

## 2 Materials and methods

### 2.1 Materials

Caffeine was purchased from Fagron GmbH & Co. KG (Barsbüttel, Germany). Methylliberine and theacrine were sourced from Nootropics Depot (Grand Rapids, Michigan, USA). Saccharin sodium was ordered from Caesar & Loretz GmbH (Hilden, Germany). TFC silicone gum type 26 was sourced from Troll Factory Rainer Habekost e.K. (Riede, Germany). The study meal components were purchased from a local supermarket: Pesto Rosso, Pesto Calabrese and Pesto Genovese were sourced from Barilla (Parma, Italy), spaghetti from Bon Pasta GmbH (Erfurt, Germany), Grana Padano from Naabtaler Milchwerke GmbH & Co. KG (Schwarzenfeld, Germany). Hydrochloric acid for the preparation of the dissolution media was purchased from Walter-CMP GmbH & Co. KG (Kiel, Germany), while sodium chloride was purchased from Sigma-Aldrich (Steinheim, Germany). All solvents used for Liquid Chromatography-Mass Spectrometry (LC-MS) analysis, i.e., water, methanol and formic acid, were ordered in LC-MS grade quality from VWR international (Fontenay-sous-Bois, France).

### 2.2 Methods

#### 2.2.1 Preparation of the ice capsules

The ice capsules were produced according to the method described by Sager et al. (Sager et al., 2018). For this purpose, a silicone base mould was filled with 0.65 mL demineralised water, sealed with the silicone upper mould and frozen at −80 °C for 15 min. After carefully removing the upper mould, the resulting ice capsules were immediately filled with a caffeine solution, theacrine solution, methylliberine solution or a mixture of all three substances at a temperature of 4 °C. The filled capsules were then frozen again at −80 °C for 15 min and subsequently sealed inside the freezer using 0.35 mL of a demineralised water solution that had been previously cooled to 4 °C, in order to prevent thawing during the sealing process. The capsules were then placed in the freezer at −80 °C for 20 min. The capsules were then stored at −20 °C. The ice capsule fillings had to be adapted compared to the original description by Sager et al. For all ice capsules, 250 mg saccharin sodium and a filling volume of 0.5 mL were used. In contrast, the amount of caffeine, methylliberine or theacrine used and the number of ice capsules applied differed between the study arms A-D. The composition of the ice capsule fillings as well as the number of ice capsules administered are shown in Table 1. Firstly, the dissolution behaviour of the ice capsules should be investigated in vitro to ensure that the substances are released quickly enough to be distributed in the ingested water and to label its gastric emptying. The ice capsules were produced one day before the respective study day. Until administration, the capsules were stored at −20 °C.

**Table 1:** Ingredients of the ice capsule filling and quantity of applied ice capsules.

Study arm	Ingredients infill	Filling Quantity (per capsule)	Number of ice capsules applied
A	caffeine	25 mg	1
	saccharin sodium	250 mg	
	demineralised water	ad 0.5 mL	
B	methylliberine	50 mg	2
	saccharin sodium	250 mg	
	demineralised water	ad 0.5 mL	
C	theacrine	50 mg	1
	saccharin sodium	250 mg	
	demineralised water	ad 0.5 mL	
D	caffeine	12.5 mg	2
	methylliberine	50 mg	
	theacrine	25 mg	
	saccharin sodium	250 mg	
	demineralised water	ad 0.5 mL	

#### 2.2.2 In vitro*-*Testing of the Formulations

The dissolution behaviour of the prepared ice capsules was investigated in a paddle apparatus (PT-DT70, PharmaTest Apparatebau AG, Hainburg, Germany). All tests were carried out in 300 mL medium at 25 °C and a stirring speed of 25 rpm. The test medium used was Simulated Gastric Fluid sine pepsin (SGFsp, pH 1.2), which was prepared according to the European Pharmacopoeia (Ph. Eur. 11). To determine the exact concentrations, 2 mL of sample volume was taken and the removed volume was replenished by adding 2 mL of SGFsp. Quantification of the analytes was performed using a validated HPLC-UV/Vis method. The key parameters of the applied method are summarized in Supplementary Tables S1 and S2.

#### 2.2.3 In vivo study

The in vivo study was performed in a cross over design with four different study arms involving twelve young volunteers aged 22–35 years (2 females and 10 males). Balancing with regard to gender distribution was not included in the study design. Inclusion and exclusion criteria were closely based on current EMA and FDA guidelines for bioequivalence studies (FDA, 2002; EMA, 2012). Female participants were only allowed to participate if they had a negative pregnancy test. Participants who had difficulty swallowing larger monolithic dosage forms were excluded from the study to avoid problems with swallowing the ice capsules. Subjects were required to provide written informed consent. The study was conducted in accordance with the Declaration of Helsinki (2013, Fortaleza, Brazil) and the Professional Code for Physicians in Germany (amended 2015 in Frankfurt, Germany). The Ethics Committee of the University Medicine Greifswald approved the study protocol and all related documents (internal registration number: BB 178/23, date of approval: 21.11.2023). The study has been registered in the German Clinical Trials Register (Deutsches Register Klinischer Studien) under the ID: DRKS00033549.

Subjects were required to abstain from caffeine-containing products for at least 72 h before ingestion of the ice capsules. Baseline levels of caffeine, theacrine, and methylliberine were kept as low as possible to avoid external influences and interactions. 10 h prior to the ice capsules administration, the subjects had to abstain from food. The consumption of caloric drinks was allowed until 3 h beforehand. In addition, non-caloric drinks were not permitted from 90 min before intake of the respective ice capsule. The study was conducted in the premises of the Centre of Drug Absorption and Transport (C_DAT) in Greifswald.

The same procedure was followed for all four study arms: The volunteers were required to provide a blank saliva sample 5 min before ingesting each formulation. Then, the ice capsules (study arm A: 25 mg caffeine, study arm B: 100 mg methylliberine, study arm C: 50 mg theacrine, study arm D: combination of 25 mg caffeine, 100 mg methylliberine and 50 mg theacrine) were administered together with 240 mL non‑carbonated water. Post-administration, subjects collected saliva samples at fixed times (4, 8, 12, 16, 20, 25, 30, 35, 40, 50, 60, 90, 120, 180, 240, 360, 480, 600 and 1440 min) over 24 h. The study protocol required the subjects to drink further 240 mL of water after 2 h. After 4 h, they were provided with a standard meal consisting of a freely chosen amount of pasta, pesto and Grana Padano and 240 mL of water. Participants were then discharged from the study unit with all restrictions lifted except for caffeine avoidance and independent saliva collection (at 360, 460, 600 and 1440 min). They were instructed to store their saliva samples in their personal freezers for the remainder of the study day. The remaining saliva samples were collected by the study staff the following day. Intensive physical activity, including exercise, was prohibited during the study. Block randomization in blocks of four was used to ensure complete counterbalancing of the sequence of study days and to minimize systematic bias. Potential inter-individual variability in the metabolism of methylliberine and theacrine was addressed through the cross over study design, in which each participant served as their own control. To further minimize the influence of known metabolic modulators, heavy smokers and habitual tea or coffee consumers were excluded, and participants were instructed to document any concomitant medications.

#### 2.2.4 Determination of salivary caffeine, methylliberine and theacrine

The subjects collected their saliva samples themselves in 2 mL SafeSeal microtubes (Sarstedt, Nümbrecht, Germany). The samples were then frozen and stored at −80 °C. For processing, the frozen saliva samples had to be thawed for 1 h at room temperature during the day of analysis. The samples were then vortexed at 4000 rpm for 30 s (IKA VORTEX 2, IKA-Werke GmbH & CO.KG, Staufen, Germany) and centrifuged for 15 min at 13000 rpm (16.060 x g) (Heraeus Biofuge Pico, Kendro laboratory products GmbH, Hanau, Germany). 100 μL of the supernatant was taken from the centrifuged saliva and transferred into 1.5 mL microtubes (Sarstedt, Nümbrecht, Germany). Subsequently, 25 μL of a solution of the internal standard (caffeine-d9, 4.0 μg/mL in acetonitrile/water (10:90 *V/V*)) was added and the microtubes were mixed again at 4000 rpm for 30 s. For protein precipitation, 200 μL of a mixture of acetonitrile with 1 % formic acid was added. The 1.5 mL microtubes were then mixed at 4000 rpm for 30 s and centrifuged at 16.060 x*g* for 10 min. From the precipitated saliva, 100 μL of the supernatant was removed again and transferred to a 1.5 mL HPLC vial into which 400 μL of a mixture of water and 1 % formic acid had previously been added. All calibration, quality control and internal standard solutions were initially prepared and dissolved in water, unless stated otherwise. Quality control (QC) solutions were then diluted 1:20 with analyte-free blank saliva. The calibration ranges were 5–1500 ng/mL for caffeine, 8–2400 ng/mL for methylliberine and 10–3000 ng/mL for theacrine.

The analysis was performed using a triple quadrupole LC-MS/MS (LCMS 8060, Shimadzu Corporation, Kyoto, Japan). The LC-MS system consisted of a SIL-40C X3 autosampler, an LC-40B X3 pump unit, a CTO-40S column oven, an SPD-40 UV detector and an FCV-20AH2 high pressure switching valve coupled to an LC-MS-8060 mass spectrometer via an electrospray ionization (ESI) unit. The stationary phase was a Phenomenex Kinetex® 2.6 μm PS C18 100 Å 150 × 2.1 mm column (Phenomenex, Torrance, CA, USA) protected with a SecurityGuard™ ULTRA cartridge (Phenomenex, Torrance, CA, USA) and attached to a SecurityGuard™ ULTRA holder (Phenomenex, Torrance, CA, USA). All eluents used were of LC-MS grade. A mixture of water with 0.1 % formic acid was used as the eluent for the inorganic phase (eluent A). The organic phase (eluent B) was methanol. The analytes were separated by a binary gradient elution (Table S3). The column oven was maintained at 40 °C, with a flow rate of 0.4 mL/min and an injection volume of 5 μL of the prepared sample. The autosampler temperature was set at 20 °C. The duration of the method was 7.50 min. The retention times of caffeine, theacrine and methylliberine were 2.45 min, 2.00 min and 3.80 min, respectively. One metabolite of methylliberine was detected after 3.30 min. By using a high-pressure switching valve, it was possible to transfer only the eluate of 1.7 min to 5.0 min to the mass spectrometer, while the rest of the eluate was collected in a waste container. The analytes caffeine, theacrine and methylliberine were detected in positive multiple reaction monitoring (MRM) mode. The detection of the metabolite was performed by single ion monitoring (SIM). An overview of the mass-spectrometric parameters can be found in Table 2. The following parameters were selected for the analysis: 300 °C ESI interface temperature, 526 °C desolvation temperature, 250 °C desolvation line temperature, 3.00 L/min nebulizing gas flow, 10.0 L/min heating gas flow and 10.00 L/min drying gas flow. An interface voltage of 4.0 kV was applied to the ESI source. The chromatograms were analyzed using LabSolutions software (version 5.97 SP1, Shimadzu Corporation, Kyoto, Japan).

**Table 2:** MRM transitions or Q3-SIM used for quantification with the corresponding collision energies.

Compound	MRM-transition (*m*/*z* → m/z)	Q3-SIM	Collision energy (eV)
Caffeine	194.90 → 138.05	–	−21
Methylliberine	225.20 → 168.25	–	−20
Theacrine	225.00 → 168.25	–	−20
Metabolite	–	211.00	–
d9-Caffeine	204.30 → 144.10	–	−20

MRM – Multiple Reaction Monitoring, SIM – Single Ion Monitoring.

The area ratio of the analytes (caffeine, theacrine, methylliberine)/internal standard (d9-caffeine) over the corresponding analyte concentration was used for calibration (linear regression, 1/ c^2^ weighting).

The method was validated in accordance with the Guideline on bioanalytical method validation of the EMA (European Medicines Agency, 2011). The method was validated for intra- and inter-day accuracy and precision at four concentration levels - QC-LLOQ (Quality Control - Lower Limit of Quantification), QC-L (Quality Control - Low), QC-M (Quality Control - Medium) and QC-H (Quality Control - High) - using six replicates within one day and 18 replicates on two separate days. The freeze and thaw stability, the long-term stability (over 12 weeks at −80 °C) and the short-term stability at room temperature (3 h) were analyzed in detail. For this purpose, quality controls with low (QC-L) and high concentrations (QC-H) were analyzed in quadruplicate. The stability after reinjection was tested for all four QC levels after 24 h in the autosampler with six replicates. Acceptance criteria for accuracy and precision were set at ±15 % for all levels of quality control, except for QC-LLOQ where a deviation of up to ±20 % was accepted. As shown in Supplementary Table S4, all validation parameters proved to be fulfilled.

#### 2.2.5 Statistics

The pharmacokinetics of the individual substances after single (study arms A, B, C) and combined administration (study arm D) were determined and compared using the saliva profiles of theacrine, methylliberine and caffeine. In order to compare the drug absorption behaviour, the profiles were deconvoluted based on the individual pharmacokinetic parameters of each subject. The two-compartment version of the Loo-Riegelman method was employed for deconvolution, using the GastroPlus® simulation software (GastroPlus® PBPK & PBBM Modelling and Simulation, Lancaster, Pennsylvania, USA). A three-compartment model was not considered as the logarithmically transformed concentration time profiles provided no evidence to support the inclusion of an additional compartment. The Wagner-Nelson deconvolution method was also evaluated, but it produced implausibly high fraction absorbed values, often exceeding 100 %. This was likely due to the rapid initial distribution processes. Therefore, the two-compartment Loo-Riegelman method was deemed the most appropriate for the dataset. The correlation of the absorption behaviour of theacrine and methylliberine with caffeine was determined on the basis of the deconvoluted data using the correlation coefficient R according to Pearson with a probability of error of alpha = 0.05. Pharmacokinetic parameters including c_max_, t_max_, AUC, and t_1/2_ were determined for each subject. T_max_ was defined as the time of maximum observed concentration for each individual. Since t_max_ is an ordinal variable and not all data sets were normally distributed, median values are reported alongside mean ± standard deviation to more accurately represent central tendency. AUC was calculated using the trapezoidal rule, and the elimination half-life (t_1/2_) was calculated from the slope of at least four terminal logarithmically transformed concentrations. In order to determine statistical differences in relation to c_max_, t_max_, t_1/2_, and AUC, only pairwise comparisons between single and combined administration were performed (A vs. D, B vs. D, C vs. D). Prior to analysis, the normality of each data set was assessed using the Shapiro-Wilk, D'Agostino & Pearson, and Kolmogorov-Smirnov tests. For data sets demonstrating a normal distribution, a paired *t*-test was employed. Non-normally distributed data were analyzed using a Wilcoxon signed rank test. The statistical software GraphPad Prism 5.0 (GraphPad Software, San Diego, California, USA) was used for analysis. If caffeine was already detectable in the blank saliva of a subject, the concentration of the first saliva sample before ingestion of the ice capsule (*t* = −5 min) was subtracted from all subsequent values for further analysis (average determination of c_max_ and t_max_, calculation of AUC). This issue occurred only sporadically in a few subjects, and in no case did the baseline concentration exceed 35 ng/mL. Details regarding the affected subjects and study arms are provided in Section 3.3.1.

## 3 Results

### 3.1 In vitro-dissolution

**Fig. 2:**
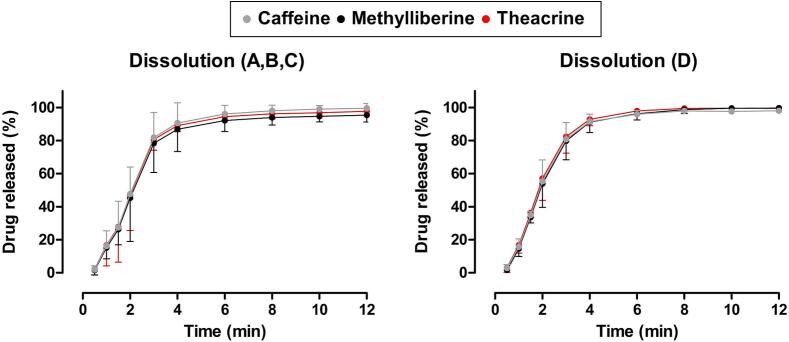
Dissolution profiles of the ice capsules filled with caffeine (A-grey), methylliberine (B-black), theacrine (C-red) or a combination of caffeine, methylliberine and theacrine (D) in 300 mL SGFsp pH 1.2 at 25 °C (*n* = 6 mean +/− standard deviation). (For interpretation of the references to colour in this figure legend, the reader is referred to the web version of this article.)

Release tests were carried out in a paddle apparatus for all different ice capsule formulations. The ice capsules comprised either 25 mg of caffeine, 50 mg of theacrine, 100 mg of methylliberine, or a combination of all three substances in the same dosages. For the release of the methylliberine ice capsules, as well as the combined formulation, the dose was divided into two capsules due to the limited size of the ice capsules to fit the whole dose. In Fig. 2, the dissolution profiles are shown. In all cases, a very rapid release of over 85 % of the substance was achieved within the first 4 min. The standard deviations obtained were greater for the release of the individual formulations than for the ice capsule, which contained a combination of all three substances.

### 3.2 Study population

All inclusion and exclusion criteria were met for the twelve volunteers recruited and no subjects were excluded from the study. All participants successfully completed the study and tolerated the study formulation well, with no adverse events or clinically relevant findings observed. In Table 3, the demographic data of the study subjects are summarized.

**Table 3:** Demographic data of the volunteers (*n* = 12).

Parameter	Median (Range)	Mean ± standard deviation
Sex	m:10, f:2	–
Age	23 (22–35)	24.2 ± 3.8
Height (cm)	183 (169–193)	181.9 ± 8.0
Weight (kg)	72 (55–89)	72.1 ± 10.5
BMI (kg/m^2^)	21.6 (18.7–24.4)	21.7 ± 2.0

m – male, f – female, BMI – body mass index.

### 3.3 Study results

#### 3.3.1 Mean profiles

**Fig. 3:**
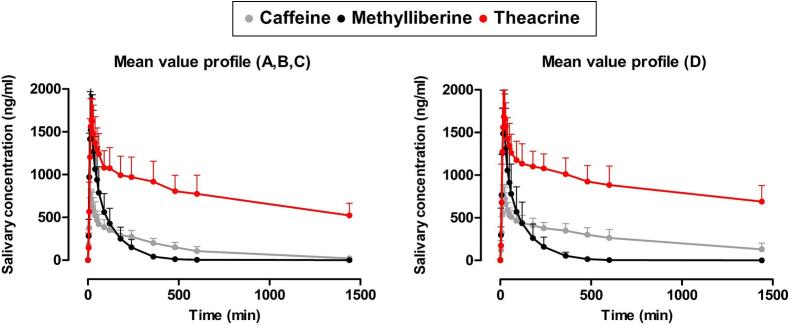
Mean salivary concentration profiles after ingestion of still water together with the ice capsules filled with caffeine (A-grey), methylliberine (B-black), theacrine (C-red) or a combination of caffeine, methylliberine and theacrine (D), (*n* = 12, mean + standard deviation). (For interpretation of the references to colour in this figure legend, the reader is referred to the web version of this article.)

The mean salivary concentration profiles of caffeine, methylliberine, and theacrine across study arms A-D are presented in Fig. 3. Individual profiles for each participant can be found in Supplementary Fig. S1–2. Noteworthy oral contamination of the oral cavity due to rapid disintegration of the ice capsules was not observed in any study participant. Participants 1 (study arm D), 7 (study arm D), 8 (study arm D), and 12 (study arms A and D) have not been completely caffeine-fasted at the beginning of the respective study day, as evident by the measured analyte concentrations in the baseline saliva samples prior to ice capsule intake. In these cases, the measured caffeine concentrations in the first baseline saliva sample were subtracted from all subsequent values.

In summary, a rapid increase in initial salivary concentrations of caffeine, methylliberine and theacrine was observed in all study arms. In all cases, maximum concentrations were reached within the first 30 min after ice capsule ingestion. It is noteworthy that after an initially similar rapid rise in concentration, the three compounds administered then diverged in their subsequent salivary profiles. While a fast decline in measured salivary concentrations of methylliberine was observed, participants still had significant levels of theacrine at the end of the study day. In Table 4, a comprehensive list of the most significant pharmacokinetic parameters is provided. It is noticeable that the individual substances differ in their half-life as well as in their overall AUC. When combined in study arm D, both caffeine and theacrine demonstrated an increase in the respective parameters, although the effect was more pronounced for caffeine. The t_max_ and c_max_ values showed only minor differences between the study arms.

**Table 4:** Overview of the pharmacokinetic parameters of 25 mg caffeine, 100 mg methylliberine and 50 mg theacrine after single (A, B, C) and combined (D) oral application (*n* = 12, mean ± standard deviation and [median]).

Parameters	Caffeine (A)	Caffeine (D)	Methylliberine (B)	Methylliberine (D)	Theacrine (C)	Theacrine (D)
c_max_ (ng/mL)	728 ± 185 [685]	796 ± 155 [783]	1725 ± 481 [1817]	1692 ± 468 [1712]	1787 ± 242 [1800]	1877 ± 296 [1838]
t_max_ (min)	19 ± 6 [16]	22 ± 7 [20]	19 ± 6 [16]	19 ± 6 [18]	23 ± 7 [20]	26 ± 13 [20]
AUC_0-tlast_ (ng*min/mL)	3367 ± 1017 [3088]	6508 ± 1945 [6430]	2496 ± 969 [2434]	2559 ± 1207 [2421]	18,631 ± 4132 [18041]	21,521 ± 4313 [20352]
t_1/2_ (h)	5.15 ± 1.21 [5.13]	15.33 ± 10.75 [12.29]	1.15 ± 0.12 [1.16]	1.27 ± 0.31 [1.28]	21.00 ± 7.55 [20.19]	27.86 ± 9.89 [26.42]

In study arms B and D, we identified one metabolite. This metabolite exhibited a mass number reduced by one methylene group (*m/z* 14) compared to methylliberine, thereby allowing its successful assignment to this substance. The t_max_ values for this metabolite were 305 ± 110 min in study arm B and 337 ± 138 min in study arm D. While a more precise quantification was not conducted due to the absence of a reference substance, further semiquantitative results can be found in Supplementary Fig. S3.

#### 3.3.2 Deconvolution of data sets

**Fig. 4:**
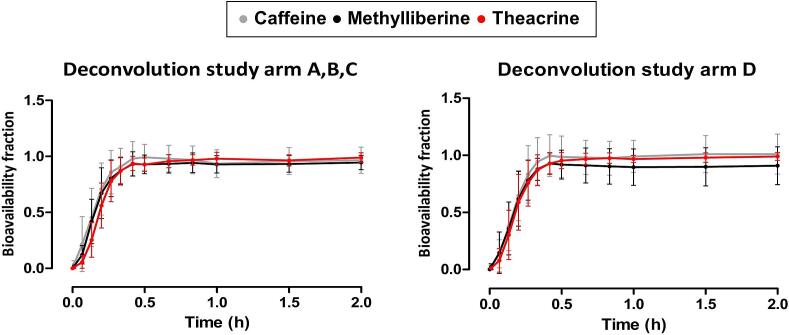
Graphical representation of the Loo-Riegelman deconvoluted data sets of caffeine (grey), methylliberine (black) and theacrine (red) after single (A, B, C) and combined (D) oral application (mean of *n* = 12 ± standard deviation). (For interpretation of the references to colour in this figure legend, the reader is referred to the web version of this article.)

As illustrated in Fig. 4, the deconvoluted datasets for all study arms (A-D) are presented, thus revealing the absorbed fraction of all three substances as a function of time. It is evident that each substance reaches nearly 100 % of its absorbed fraction (F_A_) within the first 30 min across all study arms.

The deconvoluted data of methylliberine (B, D) and theacrine (C, D) were subsequently correlated with the data of caffeine (A, D) using Pearson correlation, focusing only on the first 40 min until 100 % F_A_ was reached. For methylliberine, the correlation coefficients were *r* = 0.9973 for the single administration (B) and *r* = 0.9998 for the combined administration (D). For theacrine, correlation coefficients of *r* = 0.9865 for study arm C and *r* = 0.9977 for study arm D were determined. Consequently, the correlation between caffeine from study arm A with methylliberine and theacrine from study arms B and C, as well as the correlation for the combined administration, was found to be significant at the 0.05 alpha level. Further details can be found in Supplementary Fig. S4.

#### 3.3.3 Statistics

**Fig. 5:**
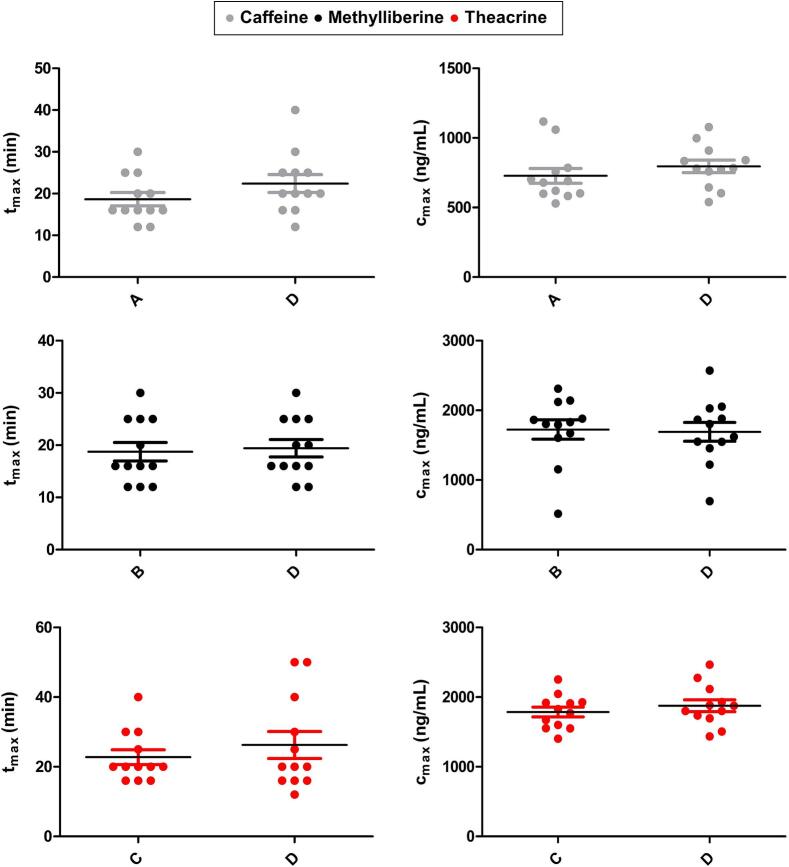
Statistical comparison of c_max_ and t_max_ of caffeine (grey), methylliberine (black), and theacrine (red) in study arms A, B, or C with study arm D. Statistical difference in c_max_ of theacrine using paired *t*-test, t_max_ and c_max_ of the other substances using Wilcoxon signed rank test, *(*p* < 0.05); **(*p* < 0.01); ***(*p* < 0.001). (For interpretation of the references to colour in this figure legend, the reader is referred to the web version of this article.)

**Fig. 6:**
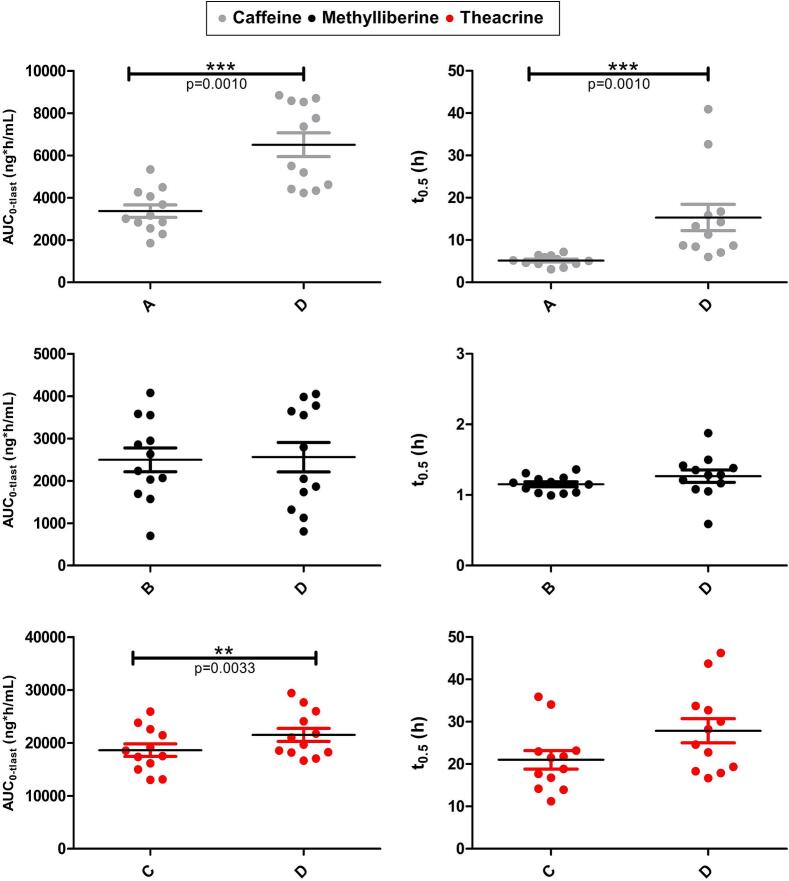
Statistical comparison of AUC_0-tlast_ and t_1/2_ of caffeine (grey), methylliberine (black), and theacrine (red) in study arms A, B, or C with study arm D. Statistical difference in AUC_0-tlast_ and t_1/2_ (*n* = 12) using the paired t-test for methylliberine and theacrine, Wilcoxon signed rank test for caffeine's AUC_0-tlast_ and t_1/2_, *(p < 0.05); **(p < 0.01); ***(p < 0.001). (For interpretation of the references to colour in this figure legend, the reader is referred to the web version of this article.)

A detailed analysis of the statistical differences between the individual substances in single (study arms A-C) and combined application (study arm D) is shown in Fig. 5, Fig. 6. As demonstrated in Fig. 5, no statistical differences in t_max_ or c_max_ can be observed for any of the three substances. As illustrated in Fig. 6, the statistical evaluation of the AUC_0-tlast_ and the half-lives is demonstrated. Statistical analysis demonstrated a significant prolongation of the AUC and t_1/2_ when caffeine was administered concomitantly with methylliberine and theacrine. Although methylliberine demonstrates no statistically significant differences following combined administration, theacrine displays a significant increase in total exposure*.* The observed prolongation of theacrine's half-life approached, but did not reach, statistical significance (*p* = 0.0584).

## 4 Discussion

In the present study, methylliberine and theacrine were intended to be quantified in saliva following oral administration in the fasted state. The absorption profiles of methylliberine and theacrine were compared to that of the established salivary marker caffeine in order to assess their potential as novel markers for determining gastric emptying of non-caloric liquids. One of the primary goals was to identify alternative marker substances that are not part of the common diet. The required fasting period would be easier to comply with for subjects and no isotopically labelled substances would have to be applied.

The methodology employed in the present study to detect marker substances in saliva offers the advantage of independent, non-invasive sampling by subjects, without the need for special equipment. Saliva has also proven to be a reliable matrix for human biomonitoring (Hofman, 2001; Caporossi et al., 2010; Michalke et al., 2015). In order to facilitate the simultaneous quantification of methylliberine, theacrine, and caffeine within this matrix, we developed and successfully validated a LC-MS/MS method. The use of specialized ice capsules aimed to protect the oral cavity from contamination by the marker substances, thereby ensuring accurate measurement of salivary concentrations. The preparation of the ice capsules proved to be an easy and reliable method, which has already been utilized in other studies for determining gastric emptying (Sager et al., 2018; Grimm et al., 2023b; Tzakri et al., 2023). The capsules contained either 25 mg caffeine, 100 mg methylliberine, 50 mg theacrine or a combination of these three substances. The doses were selected on the basis of extant literature which demonstrated the safety of the combined intake of these substances. For instance, Cintineo et al. investigated the acute co-administration of 150 mg caffeine, 100 mg methylliberine and 50 mg theacrine in healthy adults, reporting no adverse effects (Cintineo et al., 2022). Moreover, a four-week study evaluating the daily intake of 100 mg methylliberine and 50 mg theacrine found no clinically relevant effects on key health parameters such as heart rate, systolic blood pressure, high-density lipoproteins, mean corpuscular haemoglobin, basophils, absolute eosinophils, creatinine, or estimated glomerular filtration rate (VanDusseldorp et al., 2020). In the context of the present study, the administered doses did not exceed the levels that had been shown to be safe in earlier investigations (VanDusseldorp et al., 2020; Cintineo et al., 2022; Wang et al., 2020). In addition, the doses were not equated across substances because their distinct pharmacokinetic profiles result in different plasma concentrations. Therefore, the selected amounts were intended to achieve comparable exposure ranges under safe and physiologically relevant conditions.

In order to ensure that the measured salivary concentrations were exclusively attributable to the administered doses and not influenced by prior consumption, subjects were required to abstain from caffeine-containing products for a period of at least 72 h before capsule ingestion. This intervention was critical for eliminating dietary confounders, particularly in the case of caffeine, which is nearly ubiquitous in food and beverages. Furthermore, methylliberine and theacrine are present only in a limited number of specific plant species, thereby making the likelihood of basal levels in the population improbable.

The release behaviour and disintegration of the ice capsules were rapid and reproducible using a compendial apparatus. The experimental setup entailed the execution of tests in 300 mL medium at a temperature of 25 °C and a stirring speed of 25 rpm, using a USP II apparatus. While these parameters deviate from typical compendial conditions (37 °C and higher agitation rates), they were intentionally selected to simulate a worst-case scenario. These conditions were chosen to determine whether the capsules ensure sufficiently rapid release even under comparatively unfavourable conditions. Consequently, it can be inferred that the formulated capsules exhibited rapid disintegration and dissolution within the gastric environment. Based on the quick dissolution and the known first order kinetic of gastric emptying of water in the fasted state, we assumed a rapid systemic absorption which should be proportional to the measured excretion into the saliva. The basic requirement for the determination of physiological processes in saliva is a constant s/p ratio, as already shown for caffeine by Newton et al. (Newton et al., 1981). In addition, the correlation of caffeine's absorption profile with gastric emptying has been demonstrated through comparison with MRI data, as shown in the work by Sager et al. (Sager et al., 2018). Due to the high structural similarity of methylliberine and theacrine, we hypothesised a similar behaviour for these substances. To date, small, non-ionised molecules with a degree of lipid solubility and low protein binding affinity at physiological pH have proved to be most promising candidates for salivary secretion (Haeckel, 1993; Jusko and Milsap, 1993). While the secretion of caffeine into human saliva is well documented, there have been no reports of the secretion of theacrine and methylliberine into saliva until now. This study successfully demonstrated the passage of both methylurates into saliva. Based on this fundamental observation, further conclusions regarding the potential suitability of these substances can be drawn from the pharmacokinetic parameters obtained and their statistical evaluation. However, it should be noted that no established plasma/saliva ratio (s/p ratio) exists yet for methylliberine and theacrine, which is a prerequisite for drawing definitive conclusions.

The rapid rise in measured salivary concentrations and the early occurrence of t_max_ in all study arms indicated a rapid emptying of the compounds from the fasted stomach, followed by a rapid absorption. It is noteworthy that the t_max_ values (mean of *n* = 12 ± SD) of methylliberine (B: 19 ± 6 min, D: 19 ± 6 min) and theacrine (C: 23 ± 7 min, D: 26 ± 13 min) differ only marginally from those of caffeine (A: 19 ± 6 min, D: 22 ± 7 min). The statistical comparison of study arms A vs D, B vs D, and C vs D was intended to investigate whether the simultaneous use of multiple markers is generally feasible. As shown in the statistical results (Fig. 5), no significant differences were found regarding either t_max_ or c_max_. It is noteworthy that the three substances showed significant differences in their metabolic profiles. Caffeine showed a half-life of 5.15 ± 1.21 h after single administration, whereas the half-life of methylliberine was only 1.15 ± 0.12 h and that of theacrine 21.00 ± 7.55 h. Wang et al. reported similar results, with a plasma half-life of 1.5 ± 0.8 h for methylliberine and 30 ± 12 h for theacrine (Wang et al., 2020). The different half-lives of methylliberine and theacrine are associated with several practical implications. The short half-life of methylliberine enables shorter washout periods (approximately 1 day), thereby enhancing the efficacy of repetitive experimental designs. In addition, the rapid elimination of methylliberine facilitates the determination of the individual elimination rate constant (k_10_) without requiring extended sampling periods. Conversely, the longer half-life of theacrine necessitates longer washout periods (a minimum of 5–7 days), which while extending the experimental timeline, permits a more accurate assessment of the absorption process in pharmacokinetic raw data as elimination effects are less pronounced. When administered in combination, we observed a significant increase in the half-life of caffeine. The half-life of caffeine increased from 5.15 ± 1.21 h when administered alone to 15.33 ± 10.75 h when administered together with theacrine and methylliberine. A clear prolongation was also observed for theacrine, from 21.00 ± 7.55 h to 27.86 ± 9.89 h after combined administration; however, this increase fell just short of statistical significance (*p* = 0.0584), as shown in Fig. 6. The slower metabolism of caffeine and theacrine was reflected in a statistically significant increase in total exposure (AUC_₀–tlast_). Comparable results were reported by Mondal et al. and He et al. suggesting a mutual influence on the metabolism of these substances (Mondal et al., 2022; He et al., 2017). The authors hypothesised that this interaction could be explained by methylliberine reducing caffeine's hepatic clearance through the inhibition of CYP1A2, given that caffeine is primarily metabolised by CYP1A2 into paraxanthine. This interaction should be carefully considered in future study designs. In particular, the co-administration of these markers complicates the interpretation of the data obtained using AUCs. Notably, methylliberine itself is unaffected by this interaction, as no significant differences were observed in either t_1/2_ or AUC when administered in combination. Methylliberine thus emerges as an advantageous novel marker, particularly because its metabolism remains unaltered even in the presence of caffeine. This property allows accurate data interpretation without the need for caffeine abstinence prior to study procedures. Moreover, its rapid absorption and elimination further underscore methylliberine as a particularly suitable salivary marker for assessing gastric emptying, enabling shorter washout periods and more efficient study designs. Despite the metabolic differences between methylliberine, theacrine and caffeine, these differences become negligible as long as deconvolution can be performed. In this study, the Loo-Riegelman deconvolution method was utilized, as it allows for the accurate assessment of the relative amount of drug reaching the systemic circulation as a function of time. This method is particularly useful for pharmacokinetic data showing two-compartment behaviour, where it calculates the absorption-time profile by considering both central and peripheral compartments (Wagner, 1975; Nüesch, 1990). Loo-Riegelman deconvolution is advantageous because it separates the absorption and elimination phases, allowing accurate determination of absorption rates without interference from elimination kinetics. In the context of our study, once a substance enters the systemic circulation or, in our case, saliva, we aim to determine gastric emptying through its absorption profile. Although the kinetic parameters required for deconvolution are typically derived from intravenous data, this study obtained them from oral concentration-time curves generated. This approach ensured methodological consistency between the pharmacokinetic parameters and the saliva matrix. While this may result in minor uncertainties in the absolute estimates, it provides reliable information on the relative absorption kinetics under the studied conditions. In our study design, absorption was rapid, and the sampling period was long enough to allow a reliable estimate of the elimination constant. Deconvolution demonstrated uniform absorption behaviour of the compounds and validated methylliberine and theacrine as reliable markers of gastric water emptying under fasted conditions. Notably, the deconvoluted datasets exhibited a strong correlation with the caffeine data during the absorption phase (initial 40 min), underscoring the equal suitability of the three substances to evaluate gastric emptying under fasted conditions. This observation is consistent with previously published data on the gastric emptying of water in the fasted state (Mudie et al., 2014; Grimm et al., 2018).

The determination of gastric emptying of water by STT has been proven to be an effective measure, especially when the chosen dosage form disintegrates quickly in the stomach, as previously mentioned. For a substance to serve as a valid marker for gastric emptying, it is essential that gastric emptying constitutes the rate limiting step in its appearance in saliva. Subsequent processes, including absorption, and distribution must occur at a significantly faster rate to ensure the marker accurately reflects gastric emptying kinetics. The optimal scenario would be to administer the marker in the form of a liquid solution. However, since this approach would result in saliva contamination, the detection of a secondary marker in the form of a rapidly formed metabolite of the initially administered substance would represent an optimal solution. Alternatively, nasogastric administration could also be considered, but this is an invasive procedure that is unpleasant for the test subjects. Consequently, another goal of this study was the identification of new metabolites of theacrine and methylliberine. In this study, a metabolite of methylliberine was successfully detected in the saliva of study arms B and D. However, with a t_max_ exceeding 6 h in both study arms, this metabolite is deemed unsuitable for determining the rate of gastric emptying, since the appearance of the metabolite in saliva would be dominated by the rate of metabolism rather than the rate of gastric emptying. Consequently, this metabolite was not subjected to further analysis.

Overall, the methylurates methylliberine and theacrine have been identified as promising markers for determining the gastric emptying rate of water in the fasted state. Both substances showed similar absorption kinetics as caffeine, which indicates a sufficiently high solubility and permeability under comparable conditions. A significant advantage of using these substances is their secretion into saliva, which, as an easily accessible analytical matrix, simplifies the investigations.

However, for the salivary sampling technique to be applied more widely in biopharmaceutical studies, it must be confirmed that theacrine and methylliberine do not significantly influence gastric emptying or other relevant gastrointestinal parameters. The effects of caffeine on gastric emptying have primarily been demonstrated for coffee, not pure caffeine. The existent literature on this subject reports a divergence of results, with some studies reporting an acceleration of gastric emptying and others reporting a deceleration (Boekema, 1999; Lo and Akkermans, 2000; Lien et al., 1995). Nevertheless, a recent study showed that the simultaneous administration of 35 mg of unlabelled ^12^C-caffeine and 35 mg of ^13^C₃-labelled caffeine had no significant effect on gastric emptying as cross-checked with MRI, indicating that pure caffeine at this dosage does not substantially alter gastric motility (Grimm et al., 2023b). Given the rapid absorption and peak concentration of methylliberine and theacrine observed in this study, we expect these substances, like caffeine, to have little to no effect on gastric emptying or gastrointestinal function.

## 5 Conclusion

The present study demonstrated the potential of methylliberine and theacrine as alternative salivary markers for the assessment of gastric emptying of non-caloric liquids using the Salivary Tracer Technique (STT). The findings of the study indicated that both substances exhibited rapid absorption and a high degree of correlation with caffeine during the initial absorption phase. This finding thus confirmed their suitability for use in this particular application. Methylliberine, in particular, exhibited favourable pharmacokinetics, including a brief elimination half-life and negligible metabolic influence by co-administered caffeine. This approach has the potential to reduce washout periods and to facilitate comprehensive pharmacokinetic profiling within a constrained sampling window. The advantages of this approach are evident in crossover designs and multi-tracer studies. Moreover, the absence of methylliberine and theacrine in conventional diets obviates the necessity for caffeine abstinence or isotope labelling, thereby reducing participant burden and simplifying study logistics. The reliability of their salivary detectability supports non-invasive sampling without compromising analytical accuracy. In summary, methylliberin has been identified as a particularly beneficial marker for STT-based assessment of gastric emptying, enabling more efficient and participant-friendly study designs.

## CRediT authorship contribution statement

**Toni Wildgrube:** Writing – original draft, Visualization, Validation, Methodology, Investigation, Formal analysis, Data curation. **Stefan Senekowitsch:** Writing – review & editing, Validation, Formal analysis. **Robin Krüger:** Writing – review & editing, Validation, Methodology, Formal analysis. **Fabian Winter:** Writing – review & editing, Methodology. **Michael Grimm:** Writing – review & editing, Project administration, Methodology, Conceptualization. **Werner Weitschies:** Writing – review & editing, Visualization, Supervision, Project administration, Methodology, Funding acquisition, Conceptualization. **Philipp Schick:** Writing – review & editing, Supervision, Project administration, Methodology, Conceptualization.

## Informed Consent Statement

Informed consent was obtained from all volunteers in the study. Although the data do not allow the identity of the participants to be identified, written consent was obtained for the publication of this paper.

## Institutional Review Board Statement

This study was conducted in accordance with the tenets of the Declaration of Helsinki and approved by the Ethics Committee of the Greifswald University Medicine (internal registration number: BB 178/23, date of approval: 21.11.2023). This study was registered in the German Clinical Trials Register under the ID: DRKS00033549.

## Funding

No external funding was received for this research.

## Declaration of competing interest

The authors declare no conflict of interest.

## Data Availability

Data will be made available on request.

## Appendix A Supplementary data

Supplementary data Image 1
