# Supplementary material for: A comparison of theacrine and methylliberine with caffeine as salivary markers for determining gastric emptying
Source: Int J Pharm X. 2025 Nov 8;10:100442. doi: 10.1016/j.ijpx.2025.100442 (PMC12657313; doi:10.1016/j.ijpx.2025.100442)
Supplement: Supplementary file 1 — Supplementary data [file mmc1.docx]

**Table S1.** Gradient conditions of the LC-UV/Vis method used for the determination of caffeine, theacrine and methylliberine in SGFsp (eluent A: water + 0.1% formic acid, eluent B: acetonitrile).

| Time (min) | Eluent A (%) | Eluent B (%) |
| --- | --- | --- |
| 0.00 | 89.0 | 11.0 |
| 2.50 | 89.0 | 11.0 |
| 5.00 | 70.0 | 30.0 |
| 6.90 | 70.0 | 30.0 |
| 7.00 | 89.0 | 11.0 |

**Table S2.** Parameters of the LC-UV/Vis method used for the determination of caffeine, theacrine and methylliberine in SGFsp

| Parameter | Value |
| --- | --- |
| Column | Kinetex^®^ 2.6 µm F5 100 Å,  150 x 2.1 mm (Phenomenex, Aschaffenburg, Germany) |
| Column holder and cartridge | SecurityGuard™ ULTRA Holder for UHPLC columns, SecurityGuard™ ULTRA Cartridges for UHPLC-F5 column (Phenomenex, Aschaffenburg, Germany) |
| Injection volume | 5 µl |
| Flow rate | 0.4000 ml/min |
| Run Time | 9.00 min |
| Temperature of the autosampler | 20 °C |
| Temperature of the column oven | 40 °C |
| Calibration range | caffeine: 16.67-100.00 µg/mL  methylliberine: 66.67-400.00 µg/mL  theacrine: 33.33-200.00 µg/mL |
| Detection wavelength and Retention time | caffeine: 272 nm (2.80 min)  methylliberine: 283 nm (5.90 min)  theacrine: 293 nm (2.50 min) |

**Table S3.** Gradient conditions of the LC-MS/MS method used for the determination of caffeine, theacrine and methylliberine (eluent A: water + 0.1% formic acid, eluent B: methanol).

| Time (min) | Eluent A (%) | Eluent B (%) |
| --- | --- | --- |
| 0.00 | 75.0 | 25.0 |
| 5.00 | 40.0 | 60.0 |
| 5.10 | 75.0 | 25.0 |
| 7.50 | 75.0 | 25.0 |

**Table S4.** Overview of the results of the validation of the method for the determination of caffeine, theacrine and methylliberine in saliva.

| **Parameter** | **Analyte** | **QC-LLOQ** | | | **QC-L** | | **QC-M** | | **QC-H** | |
| --- | --- | --- | --- | --- | --- | --- | --- | --- | --- | --- |
| **Nominal Concentration (ng/mL)** | Caffeine | 5 | | | 12,5 | | 600 | | 1200 | |
|  | Methylliberine | 8 | | | 20 | | 960 | | 1920 | |
|  | Theacrine | 10 | | | 25 | | 1200 | | 2400 | |
| **Parameter** | **Analyte** | **Accuracy (%) (mean±SD)** | | | | | **Precision (RSD %)** | | | |
|  |  | **QC-LLOQ** | **QC-L** | **QC-M** | | **QC-H** | **QC-LLOQ** | **QC-L** | **QC-M** | **QC-H** |
| **Within-Run-Accuracy&Precision (*n*=6)** | Caffeine | 100.78±19.99 | 94.71±14.64 | 108.09±1.25 | | 109.66±1.91 | 19.83 | 13.03 | 1.16 | 1.75 |
|  | Methylliberine | 106.03±13.61 | 113.91±13.66 | 106.76±1.47 | | 108.15±0.72 | 12.83 | 11.99 | 1.37 | 0.66 |
|  | Theacrine | 106.43±14.5 | 114.31±9.33 | 109.71±1.31 | | 107.29±1.9 | 13.63 | 8.16 | 1.2 | 1.77 |
| **Between-Run-Accuracy&Precision (*n*=18)** | Caffeine | 102.43±12,58 | 99.64±11.93 | 103.19±4.64 | | 104.00±5.05 | 12.28 | 11.97 | 4.49 | 4.85 |
|  | Methylliberine | 104.35±9.83 | 109.30±10.76 | 101.60±4.35 | | 99.65±5.49 | 9.42 | 9.84 | 4.28 | 5.51 |
|  | Theacrine | 99.31±13.03 | 104.02±11.11 | 103.19±5.91 | | 102.15±4.52 | 13.12 | 10.68 | 5.72 | 4.42 |
| **Freeze-Thaw-Stability (*n*=4, 3 cycles, ‑80 °C ↔ room temperature)** | Caffeine | - | 105.82±14.86 | - | | 101.25±2.68 | - | 14.04 | - | 4.48 |
|  | Methylliberine | - | 106.55±4.23 | - | | 100.90±4.16 | - | 3.97 | - | 4.12 |
|  | Theacrine | - | 95.54±2.86 | - | | 97.38±5.01 | - | 2.99 | - | 5.15 |
| **Long-Term-Stability (*n*=4, ‑80 °C, 6 month)** | Caffeine | - | 106.59±3.37 | - | | 110.15±4.22 | - | 3.16 | - | 3.83 |
|  | Methylliberine | - | 101.73±3.75 | - | | 105.95±3.96 | - | 3.74 | - | 3.74 |
|  | Theacrine | - | 92.24±3.43 | - | | 95.14±2.62 | - | 3.72 | - | 2.76 |
| **Short-Term-Stability (*n*=4, room temperature, 4 h)** | Caffeine | - | 101.19±14.17 | - | | 106.96±3.18 | - | 14.00 | - | 2.97 |
|  | Methylliberine | - | 99.35±3.96 | - | | 98.17±1.16 | - | 3.98 | - | 3.18 |
|  | Theacrine | - | 93.35±10.19 | - | | 95.14±2.62 | - | 10.91 | - | 2.76 |
| **Autosampler Stability (*n*=6, 10 °C, 48 h)** | Caffeine | - | 105.06±8,37 | - | | 97.06±2,67 | - | 2.96 | - | 2.75 |
|  | Methylliberine | - | 101.09±1.14 | - | | 98.97±2.49 | - | 1.13 | - | 2.52 |
|  | Theacrine | - | 94.67±7.46 | - | | 89.73±3.04 | - | 7.88 | - | 3.39 |

QC – quality control, LLOQ – lower limit of quantification, L – low concentration, M – medium concentration, H – high concentration


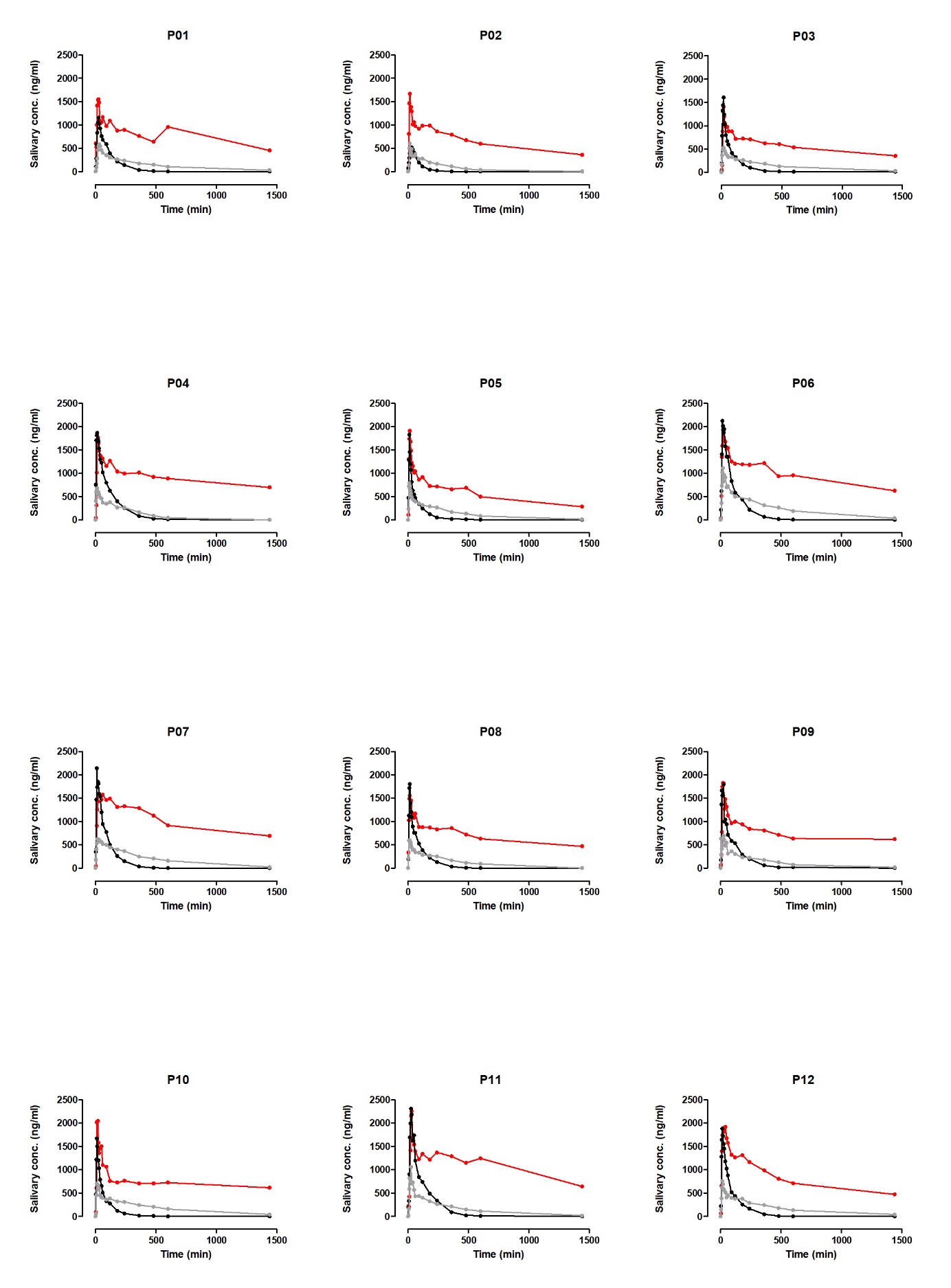


**● Caffeine A ● Methylliberine B ● Theacrine C**

**Figure S1.** Graphical representation of the individual profiles (P01-P12) following the single administration of caffeine (study arm A, grey), methylliberine (study arm B, black) and theacrine (study arm C, red).


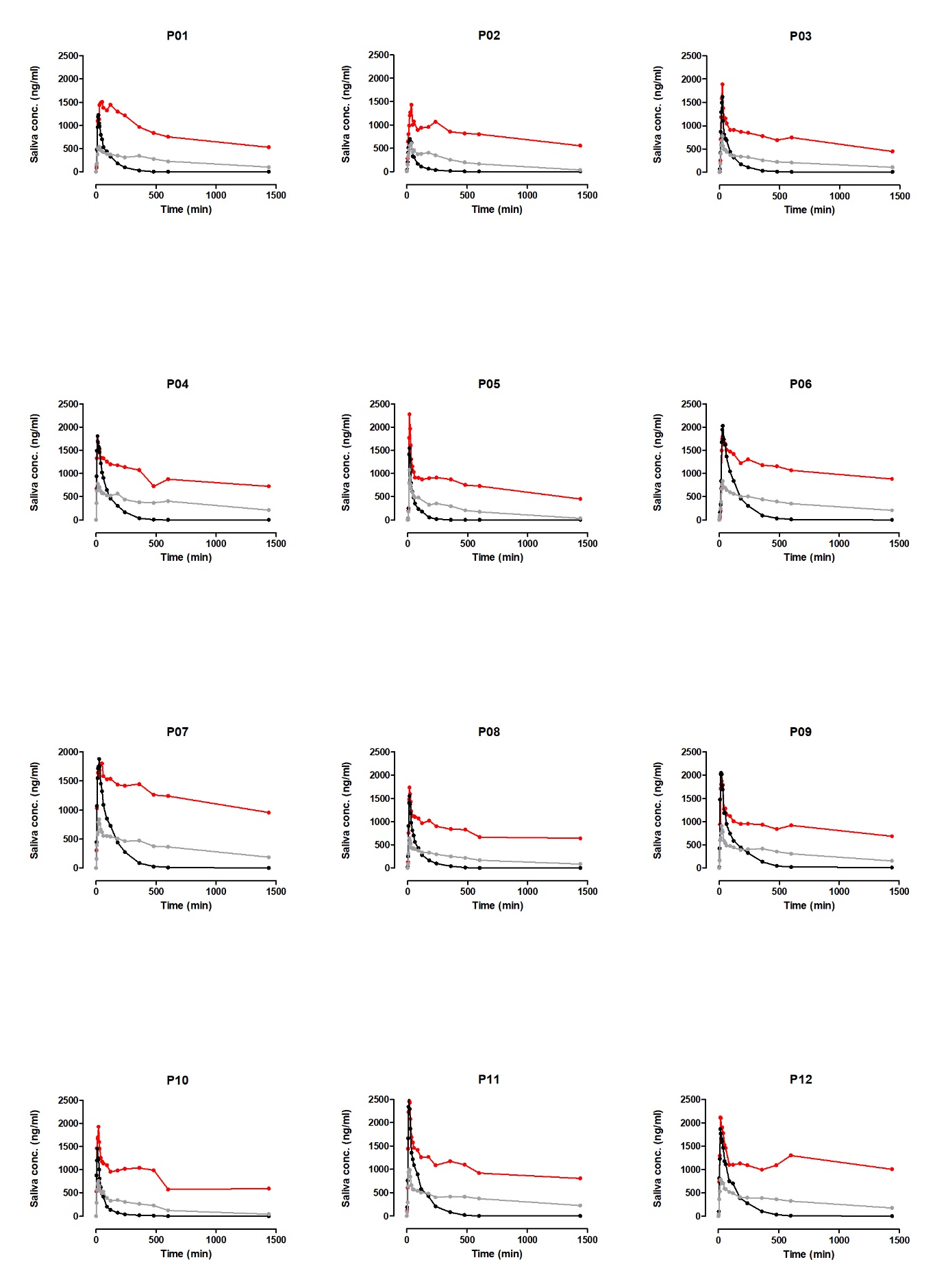


**● Caffeine D ● Methylliberine D ● Theacrine D**

**Figure S2.** Graphical representation of the individual profiles (P01-P12) following the combined administration (study arm D) of caffeine (grey), methylliberine (black) and theacrine (red).


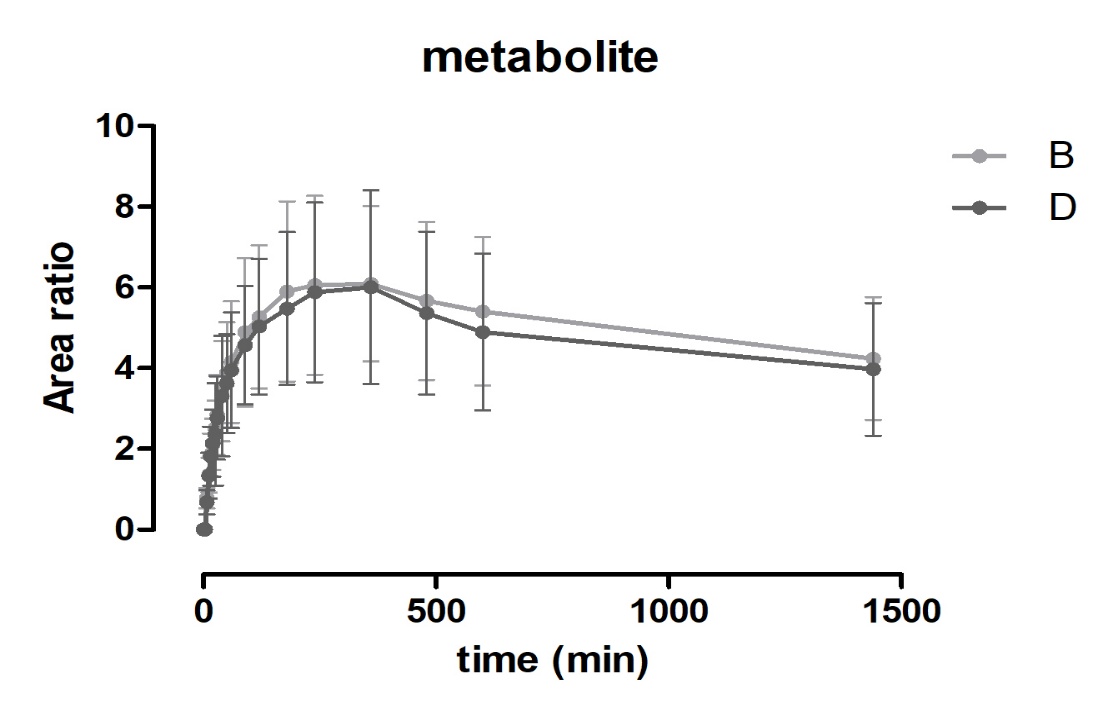


**Figure S3**. Graphic representation of the time-course a metabolite of methylliberine in saliva within study arms B (single administration of methylliberine) and D (combined application of caffeine, methylliberine and theacrine) (n=12, mean±standard deviation).


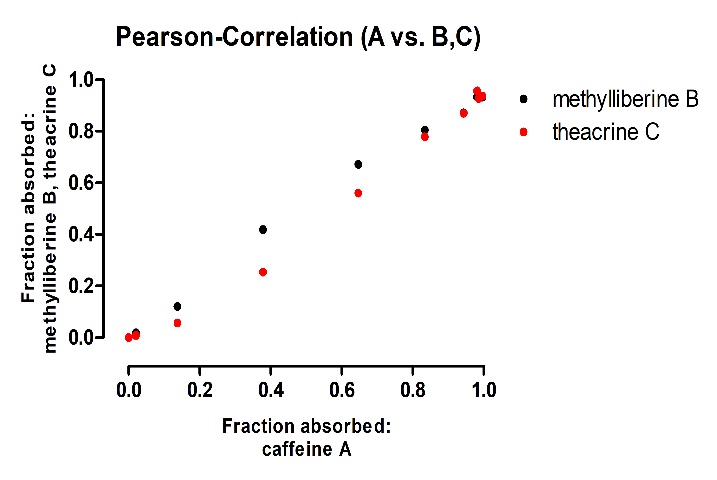

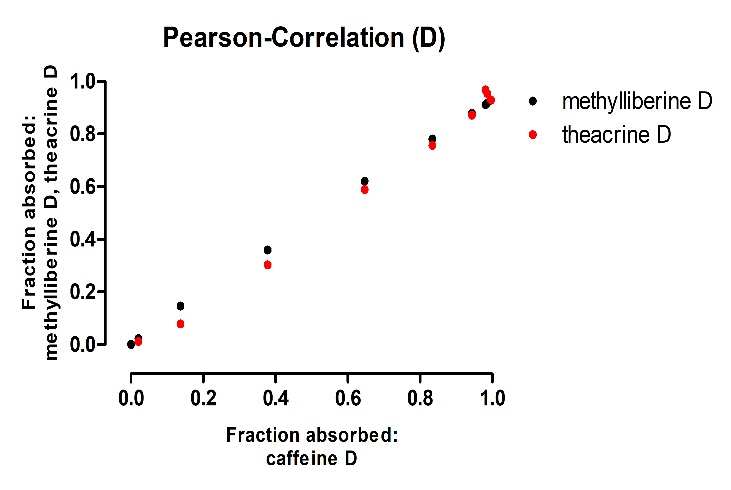


**Figure S4**. Graphical representation of the Pearson correlation of caffeine (A, D) with methylliberine (B, D) and theacrine (C, D) for alpha < 0.05
